# **Survivorship of** ****Anopheles darlingi*** (Diptera: Culicidae) in Relation with Malaria Incidence in the Brazilian Amazon*****

**DOI:** 10.1371/journal.pone.0022388

**Published:** 2011-08-08

**Authors:** Fábio Saito Monteiro de Barros, Nildimar Alves Honório, Mércia Eliane Arruda

**Affiliations:** 1 Departamento de Zoologia, Universidade Federal de Pernambuco, Recife, Brazil; 2 Laboratório de Imunoepidemiologia, Centro de Pesquisas Aggeu Magalhães, Fundação Oswaldo Cruz, Recife, Brazil; 3 Laboratório de Transmissores de Hematozoários, Departamento de Entomologia, Instituto Oswaldo Cruz-Fiocruz, Rio de Janeiro, Brazil; Tulane University, United States of America

## Abstract

We performed a longitudinal study of adult survival of *Anopheles darlingi*, the most important vector in the Amazon, in a malarigenous frontier zone of Brazil. Survival rates were determined from both parous rates and multiparous dissections. *Anopheles darlingi* human biting rates, daily survival rates and expectation of life where higher in the dry season, as compared to the rainy season, and were correlated with malaria incidence. The biting density of mosquitoes that had survived long enough for completing at least one sporogonic cycle was related with the number of malaria cases by linear regression. Survival rates were the limiting factor explaining longitudinal variations in *Plasmodium vivax* malaria incidence and the association between adult mosquito survival and malaria was statistically significant by logistic regression (P<0.05). Survival rates were better correlated with malaria incidence than adult mosquito biting density. Mathematical modeling showed that *P. falciparum* and *P. malariae* were more vulnerable to changes in mosquito survival rates because of longer sporogonic cycle duration, as compared to *P. vivax*, which could account for the low prevalence of the former parasites observed in the study area. Population modeling also showed that the observed decreases in human biting rates in the wet season could be entirely explained by decreases in survival rates, suggesting that decreased breeding did not occur in the wet season, at the sites where adult mosquitoes were collected. For the first time in the literature, multivariate methods detected a statistically significant inverse relation (P<0.05) between the number of rainy days per month and daily survival rates, suggesting that rainfall may cause adult mortality.

## Introduction

Malaria remains one of the most important infectious diseases in the world, and has reemerged in tropical regions that experience rapid population growth [1]. The understanding of age composition of anopheline mosquito populations has been considered crucial for explaining transmission and evaluating the success of control efforts [2]. The probability of survival of the vector may be the most critical factor in the transmission cycle of arthropod-borne diseases [3]–[4]. A considerable number of individuals of a given vector species must survive long enough to allow sporogonic development of *Plasmodium* spp. for it to be an efficient vector. However, little is known of age composition and survival rates of anopheline species in the Amazon region. We have reported parous rates and methods for determining parity status, as well as dispersal, of Amazonian anophelines [5]–[6]. Recently, Fouque et al. [7] have observed that peak malaria incidence in the Maroni region of French Guiana was associated with higher *Anopheles darlingi* survival rates, the most important vector in the Amazon [8]–[10] but data were not statistically significant. In this paper, we present longitudinal variations in the age structure and survivorship of *An. darlingi* in a frontier zone of the State of Roraima, in the northern Brazilian Amazon. The association of survival rates with malaria incidence was studied in detail.

## Materials and Methods

The protocol was approved by the ethics committee of the Oswaldo Cruz Foundation.

### Study site

The study was conducted within an agricultural frontier settlement of Rorainópolis, 300 km south of Boa Vista, the capital of the Province of Roraima, in the northern Brazilian Amazon. Climate and ecoregional characteristics of Roraima have also been previously reported [11]. Located deep inside the rainforest, most settlements in the area are recent (<ten years, at the time of study) and composed of multiple sideroads that run perpendicular to a main road, forming a characteristic fish-bone pattern. Malaria in the area is unstable and hypoendemic, predominantly due to *Plasmodium vivax* [12]. Sideroad 19 (00°51′N, 60°21′W) was chosen due to its higher malaria endemicity, in comparison with neighboring sideroads (Rorainópolis, Municipal Health Service, data not shown).

Sideroad 19 is a typical secondary road composed of 66 inhabited houses. Residences are spaced at 300 m intervals and lined up near the sideroad. The sideroad branches perpendicularly from the main road for 18.8 km into the rainforest.

### Climate and rainfall

In the study area, only two seasons can be distinguished, the dry season and the rainy season. A well-defined six month-long dry period lasts from November or December to April or May, with 55–60% of the precipitation occurring from May to July [13]. A pluviometer was installed in the study site for daily monitoring of rainfall throughout the collection period, from August, 2003, to July, 2004. Annual rainfall was 1367 mm/m^2^. Total monthly rainfall for the collection period is shown in Table 1. Temperature and humidity were measured by digital devices during the collection periods. Mean temperature was 28.0°C (SD = 1.11), and relative humidity was around 58% (SD = 2.88), with little yearlong variation.

**Table 1:** Total monthly rainfall (mm/m^2^), number of wet days, from August 2003–July, 2004.

Month	Rainfall (mm)	No. wet days	Cumulative malaria cases (*P. vivax*)	Mean daily temperatures (°C)
Aug	106	19	7	27.4
Sep	103	20	10	27.8
Oct	99	18	16	28.4
Nov	85	17	20	28.8
Dec	5	8	23	29.0
Jan	23	1	25	28.9
Feb	39	4	16	28.8
Mar	2	3	21	29.0
Apr	150	8	18	28.4
May	364	11	13	26.6
Jun	278	18	10	26.4
Jul	113	20	7	26.0
Total	1367	147	186	

The cumulative number of malaria cases per month diagnosed from January 2002 to December 2004 are also shown.

### Human malaria data

To explore seasonal incidences and spatial distribution, the number of cases within the period from January, 2002, to December, 2004, was analyzed. Malaria morbidity data was collected retrospectively from the Rorainópolis Municipal Health Service. Facilities were permanently available for diagnosis of cases through microscopic examination of blood smears. Only the number of patients with malaria per month was used in the study and there was no need for storage of patient information in the hospital database, which means written consent for research was not needed. Thick films were stained with 10% Giemsa solution and examined at ×1,000 under oil immersion by an expert microscopist with over 5 years of experience. Every 1–2 weeks, health agents would periodically perform thick blood smears in residents presenting non-specific acute febrile symptoms and their household contacts. All new positive malaria cases, irrespective of age, were enrolled for the study. *Plasmodium vivax* malaria cases were promptly treated with chloroquine and primaquine and *P. falciparum* with quinine or mefloquine. Cases that occurred less than 50 days after the first day of drug treatment were excluded from the study because it was unclear if they represented new cases, treatment failure or disease relapse.

### Mosquito collections

Adult collections were conducted during six bimestrial collecting excursions from August, 2003 to July, 2004, including November, 2003; January, 2004; March, 2004; and May, 2004. Captures were performed in six collecting stations, during four consecutive days. Adult mosquitoes were collected on the act of landing and identified using Consoli and Lourenço-de-Oliveira (1994) [14]. Residual spraying was performed irregularly and with large intervals between applications. Collections were not performed within three months of residual spraying. Bednets, which were unimpregnated, were used irregularly by only three families in the Sideroad. Collections were not performed in houses with bednets. Further methodological details have been previously described [15].

### Appearance of the ovarioles after oviposition and duration of the gonotrophic cycle

If the time for egg-development is known or accounted for, the approximate duration of the gonotrophic cycle, i.e. the time elapsed from one blood-feeding to the next, would vary as a function of the time elapsed from oviposition to blood-feeding. The time from oviposition to blood-feeding can be estimated by examining the condition of the terminal portions of the ovarioles, i.e. the ovariolar stalks, after passage of the eggs [2], in females in Sella's stage 1, 2 or 3 [16]. When the female oviposits, the eggs pass through the terminal portions of the ovarioles. These previously narrow conducts stretch to many times their original size to allow passage of eggs. In females that have recently oviposited (<24 h), the ovarioles still presents sac-like distensions due to this stretching. Within one day, the dilated portions return to their original size through progressive stages. It should be noted that there is no way of recognizing if the females have taken more than 24 h to find a host, since oviposition.

If egg-development is assumed to take two days [17]–[18], the duration of the gonotrophic cycle (*n*), in days, can be estimated from the ratio of mosquitoes with contracted ovariolar stalks (*c*) and the total number collected (*t*) as follows: 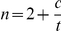. This methodology has been previously used [19]–[21] and it will be referred to, in this article, as “Charlwood's minimum cycle method” because only the minimum duration of the gonotrophic cycle can be reliably determined and cycles longer than three days cannot be detected.

Detinova (1945) [2] classified ovariolar stalk distension into five categories, from A to E, where A is the most dilated and E the most contracted. In A there is “no contraction”; B represents “first signs of contraction”; C “noticeable contraction”; in D “large dilatations” have formed; and E corresponds to “well defined dilatations”. Types A to C were considered as representing recently oviposited females. Females with A, B or C type sacs were assumed to have returned to feed on the same day they had laid eggs (a 2 day cycle) while those with type D or E dilations were assumed to have delayed one day before attempting to re-feed. Therefore, 
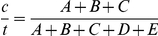
, i.e. corresponds to the number of recently oviposited *An. darlingi* females, divided by the total number of *An. darlingi* females captured in that collection period.

Data for determining gonotrophic cycle duration was collected twice, in January, 2004, and July, 2004. Gonotrophic cycle duration during other dry season collections (January and May) were considered similar to that determined in January. Cycle duration during other wet season collections (August and November) were considered similar to that determined in July.

Dissections were performed using a binocular dissecting microscope (40×) and a fiber-optics light source. Ovarian observations were performed with a binocular conventional clinical microscope (100–1,000×), in a field laboratory. Dissections were performed immediately after identification and determination of Sella's stage and only mosquitoes dissected less than six hours after the moment of capture were considered for estimating gonotrophic cycle durations.

### Data analysis: comparing ovariolar stalk dilations in different collections

A Chi-square test was also performed on the 5×2 contingency table for comparing types of ovariolar stalk dilations (A to E) in two collection periods, but categories were grouped for obtaining cells >5.

### Parous rates and survivorship

Parity was determined by checking multiple parameters, such as tracheole structure, presence of yellow bodies, presence of pedicular dilations or the appearance of the ovariolar stalks. Daily survival rates (*p*) were calculated by Davidson's method as 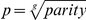, where *parity* represents the ratio between the number of parous mosquitoes and the total number of females collected, and *g* = the duration of the gonotrophic cycle in days [22]. Ninety five percent confidence intervals (95% CI) for the proportions of parous females where obtained, as proposed by Bliss (1967) [23].

### Accounting for recruitment fluctuations

Survival rates based on the proportion parous are only reliable when there is constant recruitment [22]. For example, if one increases the number of emerging adults, parous rates will tend to decrease, if mortality is kept constant. Larval density may also influence adult mosquito survival because high densities decrease pupal and adult size and fitness [24]. Concomitant larval studies verified that the quantity of larval habitats and larval densities were high during the dry season in a temporary river. On the contrary, larval densities were low during the wet season and the species apparently disappeared for one month in the river, presumably due to flushing away of larvae [25]–[26]. Meanwhile, small dams provided stable yearlong larval habitats with almost constant densities year-round [25]. In frontier zones, dams are built for creating readily available water collections, which are used for bathing, washing, recreation and fishing. They are created by blocking small streams with raised wooden and earth barriers. Apparently, predation by introduced or native fish species in these dams is not sufficient for controlling anopheline larval densities. Mosquitoes collected within 1,000 m of the temporary river were excluded from the study. This distance was chosen based on the proposed flight range of *An. darlingi* mosquitoes [5], only 4.5% of mosquitoes emerging in the river would be expected to fly more than 1000 m.

### Comparison between parous rates in different collection periods

To determine if collection periods differed significantly in the proportion of the binomial parameter “mosquito parity”, a Chi-square statistic test with Yates correction for continuity was performed using the 2×2 table describing the binomial variables number of nulliparous and parous females in each month of collection [27]. Statistical significance levels for multiple testing were adjusted using Bonferroni correction for multiple comparisons, where 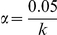 and *k* = the number of comparisons within subgroups [28].

### Density of mosquitoes of potentially dangerous age and *P. vivax* sporogonic cycle duration

Using daily survival rates, the proportion of *An. darlingi* which would survive long enough for the complete development of *P. vivax* in the mosquito can be calculated. First the duration of the sporogonic cycle, or extrinsic incubation period, must be known. This represents the time required from the moment of infection in the mosquito to complete maturation of sporozoites in the salivary glands. The time for sporogonic development of *Plasmodium* spp. in *An. darlingi* has not been specifically investigated. It is usually presumed to vary according to temperature. For *P. vivax* it would be around 10 days (range 8 to 13 days) at 27–28°C [29]. The duration of the sporogonic cycle can be indirectly estimated by the Moshkovsky method [30]–[31] which makes use of the Blunck hyperbolic equation and the Bodenheimer formula for demonstrating the relationship between temperature and gonotrophic cycle duration. In this method, first the mean daily temperatures during the period of study must be known (Table 1). The duration of the sporogonic cycle for *P. vivax* corresponds to the amount of days required for the sum of degrees Celsius above the base outdoor temperature, 14.5°C, on each day, to reach 105, the degree-days. The Moshkovsky correction factor between the mosquito resting place and the outdoor temperature was considered to be 1.0. Once the duration of the sporogonic cycle (*n*) is known, the percentage of mosquitoes surviving long enough for complete maturation of *P. vivax* can be calculated by *p^n^*. Base temperatures and degree-days for *P. falciparum* are 16°C and 111, respectively. For *P. malariae*, these figures are 16°C and 144, respectively.

*Anopheles darlingi* mosquitoes of potentially dangerous age are here defined by those females surviving long enough for complete development of *P. vivax* to an infective stage in their salivary glands (i.e. the time needed for completion of at least one sporogonic cycle). The density of dangerously aged mosquitoes (*q*) can be estimated by 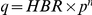, where *HBR = *human biting rate (mosquitoes per man/night). Biting densities were directly measured using the mean number of mosquitoes retrieved in 12 hour collections and are indicated in/man/night.

### Expectation of life, expectation of infective life and changes in longevity/density factors

Expectation of life was determined by 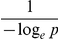 and expectation of infective life, i.e. the average number of days of infective life per infected mosquito, was determined as 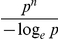 [32]. To determine the relative contributions of changes in density and longevity on the impact of residual insecticidal spraying, Garret-Jones & Grab (1964) [32] formulated the density factor (*d*) and the longevity factor (*l*). The density factor reflects how many times the density of females was affected and can be calculated as the ratio between pre and post-spraying expectations of life: 
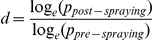
. The longevity factor is the ratio of the mosquitoes' expectation of infective lives, calculated as: 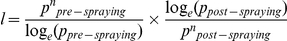. The total impact of residual insecticide spraying is given by the product *d*×*l*. We postulated that the similar principles could be used to study seasonal variations of *An. darlingi*. Since HBR is a function of the proportion of mosquitoes biting man and the total population, it will vary with breeding rates, survival rates [33] and sampling error. Assuming sampling error was small and the proportion biting man is a species-specific constant [15] we attempted to determine the relative contribution of survival and breeding by verifying how much of the density variation was due to survival rates. Survival and density are interrelated factors and if both parameters vary concurrently, an observed decrease in parous proportion will have an expected decrease in HBR [32], because the equations imply that recruitment was kept constant. A reduction in daily survival rates from 0.9 to 0.8 (exemplifying on a parasite with a 12 day sporogonic cycle), should cause a 2.11 times reduction in adult mosquito density. Field measurements can measure the amount of reduction in HBR that actually occurred: if the observed equals the expected, it would be assumed that only survival rates were affected. If the measured HBR was less than expected, it would be assumed that breeding rates were negatively affected, and if HBR is more than expected, then breeding rates and recruitment would have increased. We compared the expected decreases in mosquito density to actual decreases observed in human biting rates. Changes in the longevity factor formula had to be introduced to account for varying sporogonic cycle durations from the dry to the wet season: 
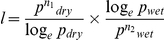
, where *n_1_* and *n_2_* are the sporogonic cycle durations in the dry and wet seasons, respectively.

### Probabilities of surviving sporogony of different *Plasmodium* spp

The probability of a mosquito surviving the duration of the sporogonic cycle varies with the *Plasmodium* spp. being considered. We determined sporogonic cycle durations for *P. falciparum* and *P. malariae*, for each collecting period with the Moshkovsky method. For comparing transmission with that of *P. vivax*, we developed a relative transmission ratio by dividing the probabilities of surviving sporogony (PSS) of each species. The ratio of “probability of surviving *P. falciparum* sporogony” divided by the “probability of surviving *P. vivax* sporogony” estimates the degree to which a mosquito population will survive to transmit falciparum malaria as compared to vivax malaria.

### Polovodova's technique

To confirm the accuracy of survival rates determined by Davidson's method, survival was estimated using multiparous dissections. We performed Polovodova's technique [2] for counting follicular stalk dilatations and determining survival rates per gonotrophic cycle. Dissections for studying follicular dilatations were performed either on the same night of capture or on the following day, in order to decrease the number of recently oviposited females. In females that have recently oviposited, i.e., types A or B ovariolar stalks where encountered, Polovodova's technique can only be applied to follicles that have degenerated in an early stage of egg development [34]. Several of these non-functional follicles had to be visualized for a good estimate to be made. Counting follicular dilatations in these recently oviposited females is difficult and occasionally could not be performed. These mosquitoes were tentatively classified as having one or two dilatations and treated as “censored data” in the survival analysis procedure. Multiparous dissections were performed in two study occasions, in January and July 2004.

### Fitting of survival times with weighted least squares fit and survival-failure analysis

Mosquitoes were grouped according to age (*x*) by counting the number of dilatations in the ovarioles and the number of specimens (*y*) in each category was determined. A regression procedure attempted fitting of the life table into four theoretical distributions (exponential, Weibull, Gompertz, and linear hazard) based on algorithms proposed by Kennedy and Gehan (1971) [35]. The logarithmic transforms of the hazard functions of all four theoretical distributions were considered linear functions of the log-transformed survival times. Fitting was performed using unweighted least squares goodness of fit, as well as two methods of weighted least squares, as proposed by Gehan and Siddiqui (1973) [36]. Fitting was evaluated by observing the Kolmogorov-Smirnoff goodness-of-fits statistics and P-values, using a significance level of α = 0.01. The presence of few categories and small sample sizes in older groups (>4 parous mosquitoes providing cell counts under 5) prevented distribution fitting using chi-square goodness-of-fit.

### Survival curves obtained with the exponential model

The fit with the highest Komolgorov-Smirnoff d statistic and P-value was the unweighted exponential model (d = 0.076; P<0.05). The exponential model was therefore adopted for further study and survival curves were obtained by a least squares fit regression of the exponential function *y* = *b*•exp(*a*•*x*), where *b* and *a* are independent parameters, as described by Service (1993) [22]. A weighted regression fit was used to account for the different sample sizes in each age group.

### Survival and failure time analysis: Kaplan-Meier product-limit method and comparison between survival curves

For survival analysis, we used the “survival and failure time analysis” module supplied in Statistica™ version 6.0 (StatSoft, Inc., Tulsa, OK). The survival function was estimated using the product-limit estimator method, as described by Kaplan and Meier (1958) [37]. A life table was created so that each time interval contains exactly one case. Multiplying out the survival probabilities across the “intervals” (i.e., for each single observation) the estimates survival function (S_(t)_) was calculated directly from the continuous survival times as the non-parametric maximum likelihood estimate using 
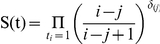
, where Π denotes the multiplication (geometric sum) across all cases less than or equal to *t*; *i* is the total number of cases, δ*(j)* is a constant that is either 1 if the *j*'th case is uncensored (complete), and 0 if it is censored.

For comparing survival curves obtained in January with July, both Breslow-Gehan's generalized Wilcoxon test and the Cox's *F* test were performed.

### Data analysis: miscellaneous tests and associations

Two-tailed Mann-Whitney U tests were used for comparing the means of variables, with the wet and dry seasons as the grouping variable. Spearman's rank correlation was used for studying the association of various variables. Forward multiple regressions were used for exploring the association of daily survival rates with meteorological variables and of log(n+1) malaria cases with adult mosquito variables. For describing pluviometry, three different parameters were compared: monthly pluviometry, the number of wet days, and degree of wetness. The number of wet days was defined as the number of days per month with more than 1 mm/m^2^. The degree of wetness was calculated by the product of monthly rainfall and total number of rainy days. Log transformations were performed when necessary for meeting assumptions on the normal distribution of residuals.

A generalized simple linear regression model was used for exploring the association of the density of dangerously aged mosquitoes with the log(n+1) malaria cases.

For modeling percentages, the logit model was preferred over linear regression, because it is not based on any assumptions regarding the variance among collections periods. Additive logistic regression modeling was used for exploring the association of the dichotomic variable parity status (nulliparous or parous output) with the number of wet days per month. Since daily survival rates are derived from a transformation of parous percentages, a logit model was also used for associating daily survival rates with the log(n+1) malaria cases.

## Results

### General results

A total of 2,193 *An. darlingi* were captured and 756 (34.47%) were dissected for determining daily survival rates and/or gonotrophic cycle duration. The number of malaria cases per month in this area is shown in Table 1. In total, 186 malaria cases of *P. vivax* malaria were reported. Only two *P. falciparum* cases were encountered and none of *P. malariae*.

**Figure 1:**
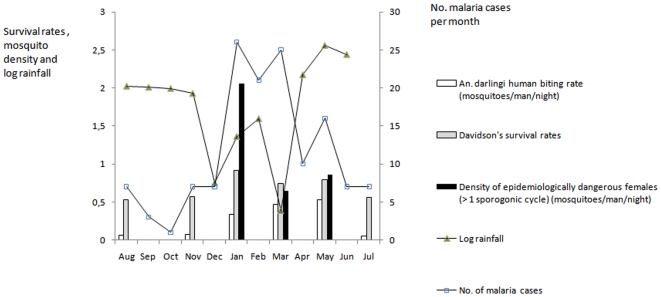
Davidson's survival rates for *Anopheles darlingi* females dissected, number of malaria cases, log rainfall, density of adult female mosquitoes and of epidemiologically dangerous mosquitoes. Data was obtained in Sideroad 19, from different collection periods, from August, 2003, to July, 2004. Number of malaria cases were obtained from January 2002 to December 2004.

Longitudinal variations of rainfall, daily survival rates, adult mosquito human biting rates, and density of dangerously aged mosquitoes are graphically shown in Figure 1. Rainfall appeared to be inversely related with daily survival rates, density of dangerously aged mosquitoes and malaria cases.

### Appearance of the ovariolar stalks after oviposition and duration of the gonotrophic cycle

The appearance of ovariolar stalks after oviposition is shown in Table 2, as well as gonotrophic cycle duration. For obtaining cells >5, grouping of categories A–C and D–E was necessary, resulting in a 2×2 table for January and July 2004. A Yates corrected chi-square test of the table determined that the two groups differed significantly (*X* ^2^ = 10.07; P<0.005).

**Table 2:** Appearance of ovariolar stalks of *An. darlingi* after oviposition determined in January and July 2004.

Appearance of ovariolar stalks	January No. (%)	July No. (%)
**A** (no contraction)	58 (50.88)	54 (80.6)
**B** (first signs of contraction)	4 (3.51)	0 (0)
**C** (noticeable contraction)	2 (1.75)	0 (0)
**D** (large dilatations formed)	1 (0.88)	0 (0)
**E** (well defined dilatations)	49 (42.98)	13 (19.4)
Total	114 (100)	67 (100)
Gonotrophic cycle duration [2+(A+B+C)/(A+B+C+D+E)]	2.43	2.19

### Parous rates and survivorship of dangerously aged mosquitoes

Daily survival rates, expectations of life, and expectations of infective life (for *P. vivax*) for *An. darlingi* in each collection period are presented in Table 3.

**Table 3:** Number of nulliparous and parous *Anopheles darlingi* females dissected in different collection periods, from August, 2003, to July, 2004.

	No. of nulliparous females dissected	No. of parous females dissected	Total dissected	Proportion parous (Bliss 95% CI)	Daily survival rates	*P. vivax* sporogonic cycle duration, in days	Probability of surviving sporogony (probability of surviving 10 days) in %	Expectation of life (expectation of infective life), in days	Mean *An. darlingi* biting density (estimated mean biting density of mosquitoes surviving >1 sporogonic cycle) in mosquitoes/man/night
**Aug**	53	17	70	0.25 (0.15–0.36)	0.524	8.14	0.52 (0.16)	1.55 (0.01)	0.72 (0.004)
**Nov**	54	24	78	0.31 (0.20–0.41)	0.571	7.34	1.64 (0.37)	1.86 (0.04)	0.84 (0.014)
**Jan**	30	114	144	0.80 (0.72–0.86)	0.911	7.29	50.41 (39.43)	10.40 (5.16)	4.08 (2.057)
**Mar**	50	47	97	0.48 (0.38–0.59)	0.742	7.24	11.54 (5.07)	3.35 (0.39)	5.64 (0.650)
**May**	48	65	113	0.57 (0.48–0.67)	0.812	8.68	13.88 (10.27)	4.39 (0.61)	6.24 (0.866)
**Jul**	185	69	254	0.27 (0.24–0.31)	0.557	9.13	0.45 (0.29)	1.68 (0.01)	0.6 (0.003)
**Total**	420	336	756						

Percent parous, Davidson's survival rates, probabilities of surviving *Plasmodium vivax* sporogony, expectations of life and mean *An. darlingi* biting densities were determined.

The sporogonic cycle durations and PSS of *An. darlingi* for the complete development of *P. vivax* varied from 0.52% in August to 50.41% in January, as shown in Table 3. The proportion of mosquitoes surviving through 10 days is also given. The mean biting density of *An. darlingi* is indicated, as well as the estimated biting density of dangerously aged mosquitoes, which varied from 0.004 to 2.057 mosquitoes/man/night in August and January, respectively.

### Grouping collections into dry and wet season categories

Yates corrected chi-square statistic test between parous rates of *An. darlingi* in different collection periods are shown in Table 4. The number of nulliparous and parous females were relatively similar in July, August and November, as deduced from the non-significant chi-square statistics between July–August, July–November and August–November. However, these differed significantly from the other three collections, i.e. January, March and May. Also March and May had similar rates among each other, but that differed from other months. January differed from all the other months. The Chi-square statistic together with the parous proportions and Bliss 95% CI permitted identification of two groups: one comprising January, March and May; and a second group August, November and July. The first group was considered as representing the dry season collections and the second group, the wet season. May was grouped as dry season presumably because collections were performed during the first half of the month, while heavy raining started mainly after the middle half of the month.

**Table 4:** Yates-corrected chi-square statistics between number of nulliparous and parous *Anopheles darlingi* in different collection periods.

	November	January	March	May	July
August	0.48	57.46**	9.05*	17.99**	0.11
November		48.35**	4.90	12.22**	0.23
January			23.29**	13.03**	97.97**
March				1.38	13.43**
May					29.79**

Significant results are indicated:

*Bonferroni corrected P<0.05;

**Bonferroni corrected P<0.001.

### Probabilities of surviving sporogony of different *Plasmodium* spp

The PSS in each collecting period, for *P. falciparum* and *P. malariae* are shown in Table 5. Also shown are the PSS ratios between these species and *P. vivax*. A PSS ratio of 1.0 would predict similar malaria transmission rates and <1.0 less efficient transmission than that of *P. vivax*. This ratio was not constant but varied along the year, reaching minimum values (less efficient transmission) in August and July. This means that variations in mosquito survival rates affect the transmission of *Plasmodium* spp. differently, according to the duration of the sporogonic cycles. The longer the sporogonic cycle of the parasite, the more it will be affected by a decrease in daily survival rates.

**Table 5:** Sporogonic cycle durations for *Plasmodium falciparum* and *P. malariae*, probability of surviving sporogony, density of dangerously aged *Anopheles darlingi* and probability of surviving sporogony ratios between these species and *P. vivax*.

	*Plasmodium falciparum*				*Plasmodium malariae*		
	Sporogonic cycle duration (days)	Probability of surviving sporogony (PSS)	Dangerously aged *An. darlingi* biting density (mosquitoes/man/night)	*falciparum/vivax* probability of surviving sporogony ratio	Sporogonic cycle duration (days)	Probability of surviving sporogony	*malariae/vivax* probability of surviving sporogony ratio
Aug	9.740	0.002	0.001	0.356	12.630	0.000	0.055
Nov	8.670	0.008	0.007	0.475	11.250	0.002	0.112
Jan	8.600	0.446	1.818	0.884	11.160	0.350	0.695
Mar	8.540	0.078	0.442	0.679	11.080	0.037	0.319
May	10.470	0.092	0.576	0.665	13.580	0.045	0.327
Jul	11.100	0.001	0.001	0.311	14.400	0.000	0.044

### Estimated and observed density factors: determining constant or variable recruitment

The longevity factor between January and July was determined as 708.63. The density factor expected by the ratio of life expectations was 6.30, which means that mosquito density was expected to have decreased 6.3 times, i.e., from 4.08 mosquitoes/man/night, in January, to 0.65 mosquitoes/man/night in July. In July, the measured mosquito density was 0.60 mosquitoes/man/night, which corresponds to a 6.80 times decrease, almost equal to the predicted value by life expectation ratios. This suggests that the observed changes in density were secondary to decreased survival rates alone and not decreased breeding. The data is consistent with no significant variation in recruitment between the two periods. The data also mean that variations in survival rates were over two orders of magnitude more important than variations in density, in reducing the capacity to transmit malaria.

### Survival curves

**Figure 2:**
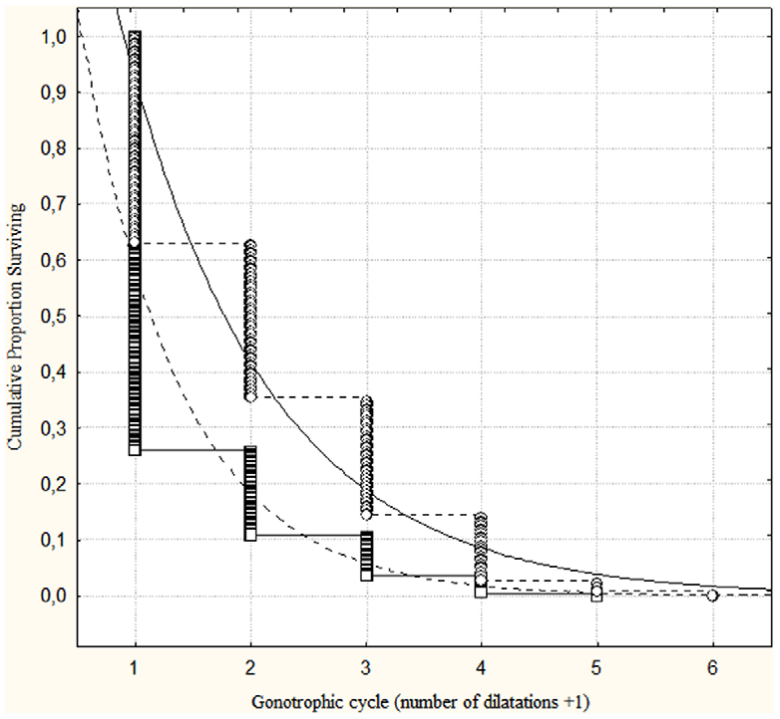
Kaplan-Meier cumulative proportions plot with weighted least squares exponential regression fitting for *Anopheles darlingi* caught in January (unbroken line and circles) and July 2004 (dotted line and squares).

The number of mosquitoes in each multiparous dissection age category is summarized in Table 6. Weighted linear regression demonstrated significant fitting of the exponential curves shown in the Kaplan-Meier diagram (Figure 2). The observed number of mosquitoes is shown as the cumulative proportion surviving and a logarithmic % survival scale was used for obtaining linear regression curves. For January, R^2^ = 0.91, F_(1, 142)_ = 755.31 (P<<0.001). For July, R^2^ = 0.97, F_(1, 248)_ = 6985.55 (P<<0.001). The regression equation, determined in January, was *y* = 4.087•e^(*x*•-0.426)^, where *x* corresponds to the number of gonotrophic cycles that have already been completed. The regression equation, determined in July, was *y* = 5.177•e^(*x*•-1.148)^. Survival rates per gonotrophic cycle and per day were determined as shown in Table 6.

**Table 6:** Number of dilatations in collections of *Anopheles darlingi* collected in January and July, 2004.

No. of dilatations	January 2004 No. (%)	July 2004 No. (%)
0	53 (30.4)	185 (71.4)
1	40 (22.9)	38 (14.7)
2	30 (17.2)	18 (6.9)
3	17 (9.8)	8 (3.1)
4	3 (1.7)	1 (0.4)
5	1 (0.6)	0 (0)
Uncountable*	30 (17.2)	9 (3.5)
Total	174 (100)	259 (100)
Survival rate per cycle (per day**)	0.65 (0.83)	0.30 (0.58)

* = Presented sac-like dilatations and counting could not be performed because abortive ovarioles were not encountered.

** = using a 2.43 and 2.19 day long cycle in January and July, respectively.

### Comparison of survival curves in each season

Cox's F-Test revealed significant differences between survival rates per gonotrophic cycle in January and July (F _(500, 288)_ = 1.92; T1 = 186.9; T2 = 207.1; P<<0.0001), with higher survival rates in January. Similarly Gehan's Wilcoxon Test was also significant (test statistic = −7.52; P<<0.0001) for differences between these two months.

### Comparison of multiparous survival curves with daily survival rates

With Davidson's method we determined daily survival rates of 91% and 55% in January and July, respectively. The survival rate for completing one sporogonic cycle (*p^n^*) corresponds to 79.1% in January and 27.1% in July, while the survival rates determined by multiparous dissections for each sporogonic cycle were 65% in January and 30% in July (Table 6).

### Forward multiple regression for associating daily survival rates with meteorological variables

To explore the association of daily survival rates with meteorological parameters, survival rates in each month were used as the dependant variable in a forward multiple stepwise regression. Independent variables were the number of mosquitoes dissected, the number of wet days in the month of collection, adult densities, monthly rainfall, degree of wetness, temperature and humidity. The only significant variable to enter the model was the number of wet days (R^2^ = 0.80; F _(1, 4)_ = 16.16; β = −0.89, SE β = 0.22; B = −0.01; P<0.05).

### Parity in wet and dry seasons

A two-tailed Mann-Whitney U test of the percent parous in the dry and rainy seasons revealed significant differences (P<0.05), with higher parous rates in the dry season.

### Spearman's rank correlation of daily survival rates with meteorological variables

Spearman's rank correlation between daily survival rates and the number of wet days was significant with non-corrected P-levels (r = −0.88; P<0.05), but only marginally significant with Bonferroni adjusted significance (P = 0.07). Spearman's rank correlation was not significant between daily survival rates and temperature (r = 0.02; Bonferroni adjusted P>1.0), humidity (r = −0.42; Bonferroni adjusted P>1.0) or degree of wetness (r = −0.25; Bonferroni adjusted P>1.0).

### Additive logistic model of parous rates and the number of wet days

Logit regression was performed using parity status as the dependant variable, the number of wet days as the independent variable, and the percent parous or nulliparous mosquitoes as the count variable. The regression equation obtained was *y* = 0.921−0.097•*x* (*X* ^2^ = 73.50 p<<0.001). This permits calculating the estimated %parous according to the number of wet days (*x*). If *x* = 30, *y* = −1.99, for example, parity can be obtained by inverting the logit equation as follows: 
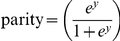
 = 0.12, i.e. a parous rate of 12%. If the number of wet days is 0, parous rates of 72% would be obtained, while 15 wet days correspond to a 37% parous rate.

### Number of malaria cases in dry and wet seasons

The number of malaria cases in the dry and wet seasons differed significantly by Mann-Whitney U test (U = 4.50; P<0.05). The number of malaria cases was higher during the dry season.

### Forward stepwise multiple regression for associating malaria cases with adult mosquito variables

A forward stepwise multiple regression was performed using the log(n+1) number of malaria cases as the dependent variable. Independent predictor variables were daily survival rate, parity ratio, adult densities, rainfall, number of mosquitoes surviving more than 10 days, temperature, and humidity. The only variable to be admitted in the model was daily survival rate (R^2^ = 0.92; F _(1, 4)_ = 23.36; β = 0.92, SE β = 0.19; B = 1.65; P<0.01).

### Association between human biting rates, daily survival rates and number of malaria cases by Spearman's rank correlation

Spearman's rank correlation between adult densities and the log(n+1) of malaria cases in Sideroad 19 was not significant (r = 0.70; Bonferroni adjusted P>0.72). But rank correlation was significant between daily survival rates and the log(n+1) of malaria cases in Sideroad 19 (r = 0.88; Bonferroni adjusted P<0.05).

### Logistic regression of malaria cases and daily survival rates

Logit regression was performed using parity status as the dependant variable, the log(n+1) number of malaria cases per month as the independent variable, and the percent parous or nulliparous mosquitoes as the count variable. The regression equation obtained was *y* = −3.344+2.851•*x* (*X* ^2^ = 73.75; P<<0.001). This means that approximately 10 cases of malaria per year (30 cases in three years) would be expected if parous rates were around 71%, but only 1 case per year if parous rates were around 16%.

### Simple regression of log malaria cases and biting density of dangerously aged mosquitoes

A simple regression between the log(n+1) of malaria cases as the dependant variable and the density of dangerously aged mosquitoes as the grouping variable showed a significant association between the two parameters (R^2^ = 0.71, F_(1, 4)_ = 9.97; P<0.05). The regression equation obtained, converted from log(n+1) cases to number of malaria cases, was the following: 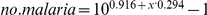, where *x* = density of dangerously aged mosquitoes. This means that a density of one dangerously aged *An. darlingi* per night would be associated with 5 cases of malaria per year in Sideroad 19 (15 in three years). For every increase in one dangerously aged mosquito per night the number of malaria cases doubles: two is associated with 10 cases per year, three with twenty etc.

### Defining the importance of adult density and daily survival rates: forward multiple regression

For differentiating adult densities from daily survival rates a forward multiple stepwise regression was performed using the log(n+1) malaria cases as the dependent variable and adult densities (human *An. darlingi* biting rate) and daily survival rates as predictor variables. A significant model (R^2^ = 0.81, F_(1,4)_ = 16.98; P<0.05) was achieved only if daily survival rates was the only variable to be admitted. Also, higher β was found with daily survival rates (β = .072) than with adult densities (β = 0.21) in the two variable marginally significant model (R^2^ = 0.82, F_(2,3)_ = 7.03, P = 0.07). This suggests that malaria incidence is better explained by daily survival rates than adult mosquito density.

## Discussion

### Gonotrophic cycle duration: literature and methodological limitations

For estimating adult survival rates, first the durations of the gonotrophic cycles must be accurately known. Limited information is available on the duration of the gonotrophic cycle of *An. darlingi* and methodological aspects have not been well defined. In Aripuanã, Mato Grosso State, Central Brazil, Charlwood & Wilkes (1979) [38], reported that only a few parous females presented unstretched ovariolar terminals, and interpreted this as indicating that the gonotrophic cycle duration required at least three days. Charlwood's minimum cycle method compared well with capture-recapture experiments in Jaru, Rondonia, Western Amazon, where Charlwood & Alecrim (1989) [20] determined that *An. darlingi* had an approximate 2.3 day oviposition cycle. The same authors also proposed a re-analysis of the data presented by Roberts et al. (1983) [17] arriving at a 2.6 day long cycle, but the data from the latter authors may have been slightly skewed because dissections were performed the following morning after collection. Our estimates of gonotrophic cycle durations for *An. darling* are within the range observed in the literature.

### Notes on the accuracy of Charlwood's minimum cycle method

Although Charlwood's minimum cycle method appears to be of limited applicability because the possibility of the real cycle being longer than the estimate, the simplicity of the method is particularly useful for repeated measures studies. The method makes the following assumptions: 1- all females with well defined dilations (type E) have returned to bite no more than 24 hours after oviposition, i.e. have gonotrophic cycles of three days; 2- there is no delay between blood-feeding and oviposition; 3- the duration of egg development is constant. To account for the possibility of longer cycles, we performed the same statistical analyses assuming that these females had returned 2, 3, 4… up to 10 days after oviposition. Longer gonotrophic cycles increase the daily survival rates during the wet season disproportionally more than in the dry season, decreasing the overall magnitude of variance between seasons. However, non-significant statistical levels for the analyses performed (P>0.05) could only be obtained if one assumed that mosquitoes with well defined dilations had taken at least 4–5 days to find a host after oviposition, i.e. had gonotrophic cycles of 6–7 days. This is a highly unlikely scenario, since the variation in the literature has ranged from 2.3 to 4.4 days for this species, with different sampling methods [7] and the longest reported *Nyssorhyncus* cycle is 5 days [39].

### On assuming constant gonotrophic cycles in longitudinal studies

To our knowledge, this is the first study to document seasonal variation in gonotrophic cycle duration, for a Neotropical anopheline. We caution that using a constant gonotrophic cycle duration for fitting parous rate data must be performed with care. It may obscure changes in breeding site availability that may occur in accordance to rainfall. However, in our study, the gonotrophic cycle variations estimated in the dry and wet seasons was not important and controlling for cycle duration with a constant parameter would not change any of the observations, since very similar results would have been obtained if any constant gonotrophic cycle from 2.5 to 4.5 days was to be used. Shorter gonotrophic cycle durations in each season could reflect the increased abundance of larval habitats in the wet season, which appears to have been more determinant than the relatively lower mean daily temperatures in the wet season (26°C, as compared to 29°C).

### Methodological limitations: spatial distribution of larval habitats; possible subtle temporal variations in gonotrophic cycle duration; and sample size considerations

We determined the gonotrophic cycle duration only twice during the year, by performing dissections soon after capture on a large number of mosquitoes. It is possible that short term temporal variations in cycle duration may have been overlooked. Also, minimal sample sizes for Charlwood's minimum cycle method and the scale of temporal variations are not yet understood. More studies are necessary to better characterize the relative role of meteorological and geographic variables and to demonstrate how cycle durations vary in time. We suggest that capture-recapture experiments or laboratory studies focusing on the time needed for ovariolar stalk contraction may be particularly instructive.

It is possible that the presence or proximity to dams may have biased the data. Dams may maintain constant recruitment because they form stable larval habitats, as compared to rivers. Adult density and survival rates near temporary rivers were not evaluated in this study. The proximity to fish-farming dams may also increase the proportion of parous to nulliparous mosquitoes, because ovipositing females tend to actively seek and concentrate at these areas [40]. The existence of spatial heterogeneity of parous rates was not evaluated in the presence study.

Sideroad 19 differs from other sideroads in the area by the quantity of dams, and this may cause variation in gonotrophic cycle duration. Fish-farming dams are known important *An. darlingi* larval habitats [41]. While 10 dams were present in Sideroad 19, neighboring sideroads had a mean of only 4.25 dams (SD = 2.2). The mean distance between fish-farms and adult collecting stations used in the study was only 110 m (SD = 26.4 m). This distance decreased by 5–10 m during the wet season. More studies are necessary to demonstrate if gonotrophic cycle durations vary in different geographical areas. We caution that parity studies that make exclusive use of parous rates, instead of multiparous dissections, should attempt to control for recruitment fluctuations. To enable this, knowledge of larval binomics in the study area is highly recommended.

### Detinova's classification

There is still incomplete knowledge of the time needed for moving between each stage of sac-like dilations after oviposition occurs. Studies have traditionally considered sacular dilation to be present or absent. In this study we arbitrarily considered types A to C as representing recently oviposited females. Confusion could arise when classifying C and D dilations as recently oviposited or not, but these represented together less than 3% of dissected females. A, B and E types accounted for the majority of mosquitoes dissected. To our knowledge, this is the first attempt to relate Detinova's sacular classification to gonotrophic cycle duration. We propose that the use of this classification may be advantageous, compared to the two category method, since it permits regression modeling and more accurate weighted means can be obtained. An accurate gonotrophic cycle determination is particularly important due to the sensibility of survival data to this estimate.

### Variation in daily survival rates appears to be related to rainfall

In our study, daily survival rates were lower during the wet season, as compared to the dry season. The best meteorological predictor of daily survival rate was the number of wet days per month. We suggest that heavy rainstorms may cause increased adult mortality by direct impact of droplets on resting or flying mosquitoes. The frequency of raining may be more important than the amount of rain, but more studies are necessary to better evaluate this. Rainfall in Southern Roraima is of the convective type, with large drop size, high rain-rates and large amounts of water per downpour, typically reaching 20 to 40 mm/m^2^ in 2–3 hours. Wind speed was not systematically measured in this study, but values from 12 to 18 km/h were common, as measured visually by the Beaufort scale, and could influence mosquito flight, dispersal [42] or even survival.

Capture-recapture experiments performed by Charlwood & Alecrim (1989) [20], at the beginning of the rainy season in that region of the Amazon, yielded daily survival rates of 83%. For the same period, we reported 81%, in May. A reanalysis of Charlwood's (1980) [21] data from Mato Grosso, during the end of the rainy season in that region, was performed. Exponential regression models using the table provided by these authors, calculated daily survival rates of 63% with Davidson's method, using the 2.3 day long cycle proposed by Charlwood & Alecrim (1989) [20]. We report 57% for the same season in Roraima. Fouque et al. (2010) [7] have also reported higher survival rates in the dry season than the wet season, in the Maroni area of French Guiana.

Studies suggest that *An. darlingi* survival rates may vary as a function of seasonal influences. Variations may occur synchronously throughout areas of the Amazon with similar rainfall patterns, but more studies are necessary to verify this.

### Methodological limitations: possible subtle temporal variations in parous rates

Parous rates were only determined once every two months. It is possible that transient variations in Davidson's parous rates, in the order of weeks, could have been missed in the study. Fouque et al. 2010 [7], reported variations around 10–30% (SD = 12–24%) from one month to the next, but the possibility of recruitment fluctuation hindered better evaluation. More studies, with smaller sampling intervals and larger samples, are needed to verify more precisely how these rates vary in time.

### Mathematical basis for comparisons of survival and density

The greater influence of parous rates in determining transmission can be explained by analyzing classic malaria modeling formulae [43]. Malaria incidence varies in a 1∶1 proportion to the density of vectors and in a 1∶1 proportion to the expectation of infective life (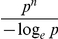). In this sense, a 16 fold increase in entomological inoculation rate could be brought about by an increase in 16 times in the man-biting rate or by an increase in 20% in survival rate [4].

### Malaria incidence was better related to daily survival rates than adult densities

Parous rates and adult densities were well correlated (0.82 correlation). This occurred because the changes observed in density were basically those expected by the decrease in survival rates, suggesting that survival, and not breeding, was the limiting factor determining density. Also, the longevity factor was much higher than the density factor and daily survival rates explained better malaria incidence than adult densities with multivariate analysis. Spearman's rank correlation of malaria cases was also significant with daily survival rates but not with density. This data suggests that malaria incidence in Sideroad 19 was better explained by daily survival rates than adult densities. To our knowledge, this paper is the first statistical study of variation of survival rates of Neotropical malaria vectors in relation to malaria incidence.

Variations in malaria transmission have been related to mosquito survival rates per extrinsic incubation period, but not survivorship per feeding cycle [44]. In the Maroni River, French Guiana, we performed a reanalysis of the entomological data collected by Fouque et al. (2010) [7] with permission of the author, comprising almost 2,700 dissections during two years. The total number of mosquitoes, the number of parous mosquitoes and the number of malaria cases were significantly higher during the dry season, as compared to the wet season, by Mann Whitney U tests (P<0.001). Spearman rank correlation was significant (P<<0.001) between the number of malaria cases in Maripasoula and the number of parous mosquitoes per month. The simple linear regression equation between the number of malaria cases and the number of parous mosquitoes was also significant (P<<0.001), although neither survival rates alone nor mosquito density alone produced significant regressions with malaria incidence. We verified that recruitment fluctuations, secondary to decreased breeding rates, were likely to have occurred because decreases in adult density were higher than that explained by decreases in survival alone. These results suggest that, in the riverine Maroni region, where there are no dams, both survival rates and density variations caused by changes in larval breeding rates may be correlated to malaria incidence. It is possible that in areas near year-round stable water collections only survival rates will be important for malaria incidence, while near small temporary rivers, where breeding may vary due to wet season flushing of larvae, both variations in survival rates and breeding will influence transmission.

Fouque et al. (2010) [7] have proposed, as an alternative to the vectorial capacity for longitudinal studies, the use of a more simplified parameter describing the number of infected mosquitoes able to transmit malaria (IMT), calculated by 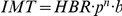, where *b* = proportion of infected mosquitoes. However, longitudinal studies frequently fail to encounter infected mosquitoes on many occasions and entolomological inoculation rates are determined with pooled data for the entire period of observation. We believe that *b* should be omitted from the equation, as was done in this study, to arrive at the simpler dangerously aged mosquitoes equation: 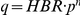.

### Wet season-predominant alluvial malaria appears to differ epidemiologically from frontier zone or highland dry season malaria

Parity studies may be particularly useful for explaining malaria incidence in areas where there is little seasonal change in anopheline biting rates. In this sense, malaria transmission in frontier zones appears to be epidemiologically distinct from alluvial malaria [45]–[46]. In frontier zones, the absence of large rivers and seasonal flooding, mean that larval habitats are relatively scarce and mosquito densities are lower. Vector breeding is dependent on the natural or artificially created water collections, being less dependent on the level of the water table. Malaria transmission occurs during the dry season [8], [46]–[47] or the dry-wet and wet-dry transitions [9], [21], [48]. Meanwhile, near large rivers, the role of female mosquito density could be more important than survival because flooding occurs during the wet season, increasing the larval population and high adult densities are obtained [49]–[51]. Longitudinal survival studies should keep this geographical heterogeneity in mind. Parous rate studies using Davidson's method may yield incorrect results near large rivers, where recruitment fluctuations are important.

### Multiparous dissections can be used to validate Davidson's daily survival rates and gonotrophic cycle durations

By comparing multiple methods of age determination in *Anopheles farauti*, Charlwood (1986) [52] suggested that rates based on multiparous dissections were more reliable than rates determined by parous dissections. This method does not require calculating gonotrophic cycle durations. For this reason, we dissected mosquitoes for obtaining survival curves. In our study, daily survival rates obtained by Davidson's method compared well to the survival curves by multiparous dissections. The good correlation between the two data sets suggests that the gonotrophic cycle durations and daily survival rates that were determined with Davidson's method were relatively accurate.

### Difficulties with Polovodova's technique

Many investigators have encountered problems while performing the Polovoda technique for age-grading [53] and Hugo et al. [54] have found that it enabled correct classification of only 57.5% nulliparous and 1, 2 or 3-parous *Ae. vigilax* females. Hoc & Wilkes (1995) [55] have proposed that ovariole sacs contract to form basal distentions which are larger than the typical dilatations. These distensions would obscure signs of the typical dilatations, i.e. previous gonotrophic activity. The typical dilatations described by Polovodova would form only when follicles degenerate at an early stage during egg development. This means that in parous mosquitoes, multiple ovarioles must be checked to find those with one or more typical dilatations, as was performed in this study. The availability of the new molecular methods may help solve the problem of an adequate age-grading technique [54].

### Duration of the sporogonic cycle of *Plasmodium* spp

The duration of the sporogonic cycle of *P. vivax* and other *Plasmodium* spp. in *An. darlingi* is poorly characterized. Although long experience with the Moshkovsky method in the former USSR has shown that it is useful for epidemiological analysis in temperate climates, we are unaware of any systematized validation with tropical malaria strains. For this reason, when we report the estimated percent of mosquitoes surviving enough to transmit malaria, both the percentage that would survive one or more cycle, as estimated by the Moshkovsky method, as well as the percentage that would survive 10 days is given, to show the results if temperature-dependant changes in sporogonic cycle duration were ignored. Relatively similar data were obtained with either method, with more dangerously aged mosquitoes in the dry season. However, the density of dangerously aged mosquitoes was better associated with malaria incidence than the 10 day old densities. For the latter parameter, the simple linear regression with log(n+1) malaria was not significant (P>0.1).

### Probability of surviving sporogony ratios and duration of the sporogonic cycles

The duration of the extrinsic incubation periods of *Plasmodium* spp. are, in decreasing order: *P. malariae*>*P. falciparum*>*P. vivax*. Longer cycles decrease the probability that a mosquito will survive sporogony. For every day increase in extrinsic incubation cycles the probability of surviving sporogony decreases by *p^n^* ^+1^. *Plasmodium* spp. with longer extrinsic incubation periods, such as *P. falciparum* and *P. malariae*, were more affected by seasonal decreases in daily survival rates than *P. vivax*. This is compatible with the prevalence of *Plasmodium* spp. in Sideroad 19, where only two cases of *P. falciparum* malaria were detected, both in the dry season, and no cases of *P. malariae*, based on thick smears only. These results were not confirmed with Polymerase Chain Reaction (PCR) and asymptomatic subjects were not regularly sampled.

Hypoendemic frontier malaria in the Amazon, the second colonization phase of frontier malaria [45], is composed mainly of *P. vivax*, followed by *P. falciparum*. Cases of *P. malariae* are considered to be relatively uncommon, when only thick smears are analyzed. We postulate that the more efficient transmission of *P. vivax* could be secondary to a vector survival-dependent limiting factor for disease transmission, compatible with sporogonic cycle durations of *Plasmodium* spp. It is possible that performing effective insecticide spraying increases the *P. vivax* to *P. falciparum* malaria ratios. The relative prevalence of *Plasmodium* spp. in the human population may also aid in identifying localities where mosquito survival is the limiting factor in disease transmission, but more studies are necessary to verify this.

### Survival curves and constant versus age-dependant survival

Fitting of the exponential model of survival curves initially suggest constant mortality rates in the different age groups of the mosquito population, rather than age dependant survival rates. However, studies with larger samples are needed to verify this and the existence of age-dependant survival rates is possible. Age-dependant survival would be suggested if the data fitted better a Gompertz [56] or a Weibull distribution rather than an exponential distribution. If mortality is, in fact, age-dependant, models will be more complex, *p* cannot be regarded as a constant, mathematical equations must be adapted and values recalculated. Age-independent models tend to overestimate the transmission potential of older mosquitoes, overestimating vectorial capacity [57].

### Final comments

Our results indicate that survival rates of *An. darlingi* may vary in accordance to rainfall and the wet season could be associated with lower adult survival rates, resulting in decreased malaria transmission. More studies are necessary to better evaluate the influence of meteorological parameters with survival rates.

In small temporary rivers, *An. darlingi* breeding may be limited to the dry season [25]–[26]. Rainfall may cause larval mortality in small rivers by the following mechanisms: flushing out larvae with strong currents, direct impact of raindrops on the water, depending on the size of raindrops; ejection of immature stages onto muddy surroundings; and exhaustion of larvae by constantly moving away from the surface to avoid being struck by raindrops [58]. Malaria transmission in this setting would be limited by both mosquito survival and breeding rates. But the construction of small dams, blocking small waterways, may enable breeding throughout the year [25]–[26]. Dams permit constant recruitment of relatively small densities of *An. darlingi* and Barros *et al* (2011) [26] have proposed that the species prefers areas with obstructions to river flow, decreased luminance (shade) and proximity to human dwellings. Malaria transmission in this setting would be basically limited by mosquito survival alone. It appears that anthropic modification of the environment may remove the natural balance of limiting factors such as river currents caused by heavy raining [58]. Determining key transmission factors may help direct control efforts. If survival is the limiting factor, residual insecticide spraying would be particularly useful. If larval and/or adult density is determinant, larvicidal methods or increasing the distance between larval habitats and humans should be favored.
